# Prediction of clinical diagnosis of Alzheimer’s disease, vascular, mixed, and all-cause dementia by a polygenic risk score and *APOE* status in a community-based cohort prospectively followed over 17 years

**DOI:** 10.1038/s41380-020-0764-y

**Published:** 2020-05-13

**Authors:** H. Stocker, L. Perna, K. Weigl, T. Möllers, B. Schöttker, H. Thomsen, B. Holleczek, D. Rujescu, H. Brenner

**Affiliations:** 1 Network Aging Research, Heidelberg University, Heidelberg, Germany; 2 Division of Clinical Epidemiology and Aging Research, German Cancer Research Center, Heidelberg, Germany; 3 Medical Faculty, Heidelberg University, Heidelberg, Germany; 4 Department of Translational Research in Psychiatry, Max Planck Institute of Psychiatry, Munich, Germany; 5 Genewerk GmbH, Heidelberg, Germany; 6 Saarland Cancer Registry, Saarbrücken, Germany; 7 Department of Psychiatry, Psychotherapy and Psychosomatics, University of Halle, Halle, Germany

**Keywords:** Genetics, Diseases

## Abstract

The strongest genetic risk factor for Alzheimer’s disease (AD) is the ε4 allele of Apolipoprotein E (*APOE*) and recent genome-wide association meta-analyses have confirmed additional associated genetic loci with smaller effects. The aim of this study was to investigate the ability of an AD polygenic risk score (PRS) and *APOE* status to predict clinical diagnosis of AD, vascular (VD), mixed (MD), and all-cause dementia in a community-based cohort prospectively followed over 17 years and secondarily across age, sex, and education strata. A PRS encompassing genetic variants reaching genome-wide significant associations to AD (excluding *APOE*) from the most recent genome-wide association meta-analysis data was calculated and *APOE* status was determined in 5203 participants. During follow-up, 103, 111, 58, and 359 participants were diagnosed with AD, VD, MD, and all-cause dementia, respectively. Prediction ability of AD, VD, MD, and all-cause dementia by the PRS and *APOE* was assessed by multiple logistic regression and receiver operating characteristic curve analyses. The PRS per standard deviation increase in score and *APOE4* positivity (≥1 ε4 allele) were significantly associated with greater odds of AD (OR, 95% CI: PRS: 1.70, 1.45–1.99; *APOE4*: 3.34, 2.24–4.99) and AD prediction accuracy was significantly improved when adding the PRS to a base model of age, sex, and education (ASE) (c-statistics: ASE, 0.772; ASE + PRS, 0.810). The PRS enriched the ability of *APOE* to discern AD with stronger associations than to VD, MD, or all-cause dementia in a prospective community-based cohort.

## Introduction

The etiology of Alzheimer’s disease (AD), the most prevalent form of dementia, remains poorly understood, although it is evident that genetic predisposition plays a fundamental role [1]. The heritability of late-onset AD has been estimated as high as 79% [2]. The ε4 allele of Apolipoprotein E (*APOE4*) is the strongest known genetic risk factor of late-onset AD, but only 7% of dementia cases are attributable to *APOE4* [3], suggesting that additional genetic or environmental factors are of high relevance in AD pathogenesis [4]. In recent years large-scale genome-wide association studies (GWAS), including meta-analyses with up to 94,437 AD cases, have identified and confirmed many more genetic loci associated with AD beyond *APOE4* [5–7].

In order to collectively consider the relatively small effects of the individual genetic loci, the development of polygenic risk scores (PRSs) for AD has greatly advanced [8]. The results have illustrated that genetic risk, as measured by the PRSs, was consistently significantly associated with AD [8], although disease prediction accuracy was quite varied (c-statistic range: 0.57–0.84) [9–16]. The majority of previous studies have examined the PRS in a case-control study design in a sample within or associated to a previous genome-wide association (GWA) meta-analysis, the International Genomics of Alzheimer’s Project (IGAP), from which the associated genetic variants included in the PRSs were derived [5, 8].

The combination of *APOE4* presence and PRS classification presents a genetic risk stratification strategy that may be beneficial for future use in therapeutic development research and precision medicine. A more complex genetic risk stratification strategy could provide more specific information, which could become critical in individualized therapeutics [17, 18]. However, AD rarely occurs in isolation [19] and the relationship between AD genetic risk and other dementias could better inform risk stratification and the specificity of AD genetic risk.

To our knowledge a PRS for AD has not been evaluated in a prospective community-based cohort completely independent of the IGAP consortia or used the most recent AD GWAS meta-analyses data, and the association to vascular and mixed dementia (VD/MD) has yet to be explored. The aim of this study was to build upon previous work by calculating a PRS utilizing AD associated single nucleotide polymorphisms (SNPs) from the largest GWA meta-analysis to date, and to evaluate the score’s prediction of clinical diagnosis of AD, VD, MD, and all-cause dementia within a large community-based cohort study followed over 17 years. A secondary aim of this study was to investigate the PRS and *APOE4* across age, sex, and education strata.

## Methods

### Study design and population

The PRS was derived from the most recent IGAP meta-analysis data [7] and applied in a prospective population-based cohort, the ESTHER study, followed over 17 years [20, 21].

Summary statistics from stage 1 of the IGAP meta-analyses from Kunkle et al. were utilized [7], in which genotyped and imputed data on 11,480,632 SNPs was used to meta-analyze four previously published GWAS consortia datasets consisting of 21,982 AD cases and 41,944 controls (The Alzheimer Disease Genetics Consortium; The European AD Initiative; The Cohorts for Heart and Aging Research in Genomic Epidemiology Consortium; and The Genetic and Environmental Risk in AD Consortium Genetic and Environmental Risk in AD/Defining Genetic, Polygenic and Environmental Risk for AD Consortium (GERAD/PERADES)) [7].

The subjects for the analyses for this study are drawn from the ESTHER study, a large population-based cohort study conducted in Saarland, Germany [20, 21]. A total of 9940 participants aged 50–75 years attending a general health examination were recruited by their general practitioners (GPs) in a statewide study in Saarland, Germany in 2000–2002. A general health examination is offered at no cost to the patient every two years to adults aged 35 and older in the German health care system. Participants completed standardized self‐administered health questionnaires and provided blood samples, which were stored at −80°. Information regarding age, sex, education, medical history, and lifestyle factors was collected at baseline through participant questionnaires and medical records. Follow‐up questionnaires, medical records, and biological samples were collected after 2, 5, 8, 11, 14, and 17 years. The ESTHER study was approved by the Ethics Committee of the Medical Faculty of Heidelberg University and of the Physicians’ Board of Saarland, and all participants gave written informed consent.

**Fig. 1:**
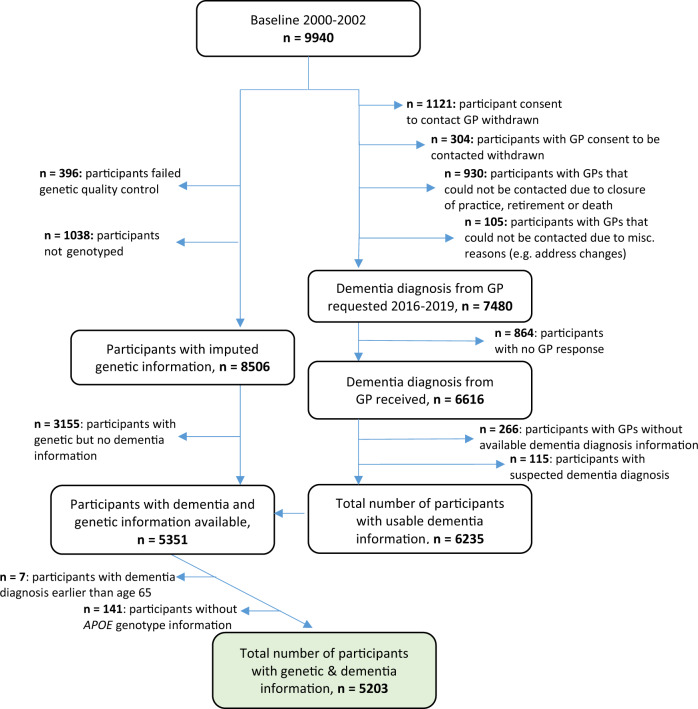
Flow chart of ESTHER study participants in the analyses.

AD, VD, MD, and all-cause dementia diagnoses were collected from participants’ GPs during the 14-year and 17-year ESTHER follow-ups as previously reported [22]. Briefly, GPs of all ESTHER participants were contacted at the 14-year and 17-year follow-ups and asked to fill out a detailed questionnaire regarding dementia diagnoses of their patients as well as to provide all available medical records of neurologists, psychiatrists, memory, or other specialized providers. The 17-year follow-up was still pending a response from a second mailing of the dementia questionnaire to those GPs who had not yet responded to the initial mailing at the time of this publication. The current guidelines in Germany for AD diagnosis follow the National Institute on Aging and the Alzheimer’s Association [23] or the International Working group (IWG)-2 criteria [24, 25], for VD diagnosis the National Institute of Neurological Disorders and Stroke-Association Internationale pour la Recherche et l’Enseignement en Neurosciences criteria [26], and for MD diagnosis the IWG-criteria for mixed dementia [24, 25]. All-cause dementia diagnoses are recommended if the dementia symptoms outlined by the ICD-10 are present for at least 6 months [25, 27]. Participants with dementia diagnoses before the age of 65 (*n* = 7) and those that did not have *APOE* genotyped information (*n* = 141) were excluded. Overall, 5203 participants with available genotyping and dementia information were included in this study (Fig. 1).

### Genotyping and imputation

*APOE* was determined based on allelic combinations of the SNPs rs7412 and rs429358 using predesigned TaqMan SNP genotyping assays (Applied Biosystems, Foster City, CA). Genotypes were analyzed in an endpoint allelic discrimination read using the Bio‐RAD CFX Connect System (Bio‐Rad Laboratories, Hercules, CA).

DNA genotyping has been previously described elsewhere [28]. Briefly, blood samples were taken during a routine health examination and stored at −80 °C until analysis. DNA from whole blood samples was collected using a salting out procedure. The extracted DNA from blood cells was genotyped using the Illumina Infinium OncoArray and Global Screening Array BeadChips (Illumina, San Diego, CA, USA).

General genotyping quality control assessment was done following the Nature Protocols article from Anderson et al. [29]. Imputation of the quality controlled data was conducted using the Michigan Imputation Server, where SHAPEIT2 was used to phase the data, and MiniMac 4 was used to impute to the HRC Version r1.1 2016 reference panel [30, 31].

### Polygenic risk score calculation

The PRS in this study was a weighted score including AD associated SNPs, calculated by summing the number of risk alleles weighted by the magnitude of association (ln of the odds ratio (OR)) from Kunkle et al. [7].

Using summary statistics from Kunkle et al., SNPs reaching genome-wide significance in the IGAP meta-analysis were extracted from the imputed ESTHER data, which resulted in 1234 SNPs extracted. Linkage disequilibrium-based clumping was carried out, providing the most significantly associated SNP in each region of linkage disequilibrium (using PLINK clumping command with a pairwise *r*^2^ threshold of 0.2). After linkage disequilibrium-based clumping, 106 SNPs remained. Then, SNPs within or directly upstream/downstream from the *APOE* locus (chr19: 45,404,000–45,418,000) were excluded (*n* = 9). Finally, a minor allele frequency (MAF) threshold of 0.01 was applied that resulted in an additional 25 SNPs excluded. The remaining included SNPs had imputation quality median *R*^2^ = 0.92 (*R*^2^ range: 0.47–0.99). A total of 72 SNPs were included in the PRS (Supplementary Table 1).

The score was normalized by subtracting the mean and dividing by the standard deviation (SD), which were both calculated from the overall sample. For the sake of comparability of prediction performance of PRS and *APOE*, the cutoff for PRS+ was determined as the score point in which the proportion of PRS+ individuals was equal to the proportion of *APOE4*+ (≥1 ε4 allele) individuals in the control group. It should be noted that this not a true or validated threshold but was chosen for comparability with *APOE* only.

### Statistical analyses

Descriptive statistics were calculated to provide information on participant characteristics, while chi-square and *t*-tests were completed comparing both AD, VD, MD, and all-cause dementia cases to individuals without dementia diagnosis. Multivariable logistic regression models with 95% confidence intervals (CI) were used to assess differences in outcome as OR between dementia cases and individuals without dementia diagnosis based upon the PRS and *APOE4* status. The PRS was considered per SD increase in score, as quartiles, and as a binary variable following the cutoff previously described. *APOE* status was utilized as a categorical variable based upon allele type/count (*APOE* ε3ε4, ε4ε4 vs. ε3ε3) and as a binary variable (*APOE4*+: ≥1 ε4 allele vs. *APOE*-: no ε4 allele). In addition, PRS and *APOE4* status were combined and ORs were calculated for individuals that were both PRS+ and *APOE4*+ compared with the reference PRS− and *APOE4*−. Covariates for all logistic regression analyses included age, sex, ten principal components, and education, measured by years of formal education (≤9, 10–11, ≥12 years; standard categories of the German school system; the lowest category corresponds to a leaving certificate from school, the highest category corresponds to qualification for university). Stratified analyses and interaction testing for age, sex, and education by PRS, *APOE4*, and PRS & *APOE4* status together were computed for all outcomes. Multiple imputation (*n* = 5) for education covariates missing at random was carried out following the Markov chain Monte Carlo (MCMC) method [32].

Receiver operating characteristic (ROC) curve analysis was completed for both the PRS and *APOE*, where the PRS was considered continuously and *APOE* was considered categorically (*APOE* ε2ε2, ε2ε3, ε3ε4, ε4ε4 vs. ε3ε3). For AD, VD, MD, and all-cause dementia, ROC curves were calculated based upon: (1) age, sex, and education; (2) age, sex, education, and PRS; (3) age, sex, education, and *APOE*; and (4) age, sex, education, PRS, and *APOE*. ROC contrast analysis using the DeLong test was conducted to compare for significant differences between curves [33].

All statistical analyses were two-sided, conducted at an α-level 0.05, and completed using SAS software, version 9.4 (SAS institute, Cary, NC).

## Results

### Participant characteristics

The participants from the ESTHER study that had both genotyping and dementia information available for these analyses included 103 AD, 111 VD, 58 MD, 359 all-cause dementia cases, and 4844 participants without dementia diagnosis. Seven participants had dementia diagnoses before the age of 65 and were therefore excluded. The mean length of follow-up of all included participants was 14.4 years. Main characteristics of study participants are shown in Table 1 and additional *APOE* characteristics in Supplementary Table 2. The mean age at baseline in AD cases was 67 years, VD/all-cause dementia cases 68 years, MD cases 69 years, and participants without dementia diagnosis 61 years. The mean age of diagnosis was 77, 79, 79, and 78 for AD, VD, MD, and all-cause dementia cases, respectively. All groups included slightly more females (51–59%) than males. In all groups the majority of individuals completed 9 years of formal education or less (83% AD cases, 78% VD cases, 80% MD cases, 81% all-cause dementia cases, and 72% participants without dementia). PRS positivity was evident among half (52%) of AD cases, 35% of VD cases, 31% of MD cases, 38% of all-cause dementia cases, and a quarter (25%) of participants without dementia diagnosis. *APOE4* positivity was evident among half (51%) of AD cases, 37% of VD cases, 35% of MD cases, 40% of all-cause dementia cases, and a quarter (25%) of participants without dementia diagnosis.

**Table 1:** Participant characteristics—ESTHER cohort study.

	Alzheimer’s disease	Vascular dementia	Mixed dementia	All-cause dementia	Participants without dementia	*p* value^1^	*p* value^2^	*p* value^3^	*p* value^4^
*n*	103	111	58	359	4 844				
Age at baseline, mean ± SD	67.2 ± 4.7	67.6 ± 4.7	68.7 ± 4.2	67.6 ± 4.8	61.2 ± 6.4	<0.0001	<0.0001	<0.0001	<0.0001
50–64 years at baseline	30 (29.1)	27 (24.3)	10 (17.2)	95 (26.5)	3250 (67.1)	<0.0001	<0.0001	<0.0001	<0.0001
65–75 years at baseline	73 (70.9)	84 (75.7)	48 (82.8)	264 (73.5)	1594 (32.9)				
Age at diagnosis, mean ± SD	76.9 ± 4.6	78.8 ± 5.1	78.9 ± 4.8	78.2 ± 5.1	–	–	–	–	–
Female, *n* (%)	60 (58.2)	59 (53.2)	34 (58.6)	182 (50.7)	2626 (54.2)	0.42	0.83	0.05	0.20
Male, *n* (%)	43 (41.8)	52 (46.8)	24 (41.4)	177 (49.3)	2218 (45.8)				
≤9 years education, *n* (%)	85 (83.3)	83 (78.3)	44 (80.0)	279 (80.9)	3431 (72.4)	0.03	0.40	0.46	<0.01
10–11 years education, *n* (%)	8 (7.8)	12 (11.3)	6 (10.9)	32 (9.3)	718 (15.2)				
≥12 years education, *n* (%)	9 (8.8)	11 (10.4)	5 (9.1)	34 (9.8)	586 (12.4)				
PRS+, *n* (%)	53 (51.5)	39 (35.1)	18 (31.0)	135 (37.6)	1211 (25.0)	<0.0001	0.02	0.29	<0.0001
PRS−, *n* (%)	50 (48.5)	72 (4.9)	40 (69.0)	224 (62.4)	3633 (75.0)				
PRS Q1	19 (18.4)	29 (26.1)	18 (31.0)	81 (22.6)	1221 (25.2)	<0.0001	0.01	0.47	<0.0001
PRS Q2	15 (14.6)	16 (14.4)	14 (24.2)	69 (19.2)	1243 (25.7)				
PRS Q3	16 (15.5)	27 (24.3)	10 (17.2)	77 (21.4)	1213 (25.0)				
PRS Q4	53 (51.5)	39 (35.1)	16 (27.6)	132 (36.8)	1167 (24.1)				
*APOE4*+, *n* (%)	52 (50.5)	41 (36.9)	20 (34.5)	143 (39.8)	1216 (25.1)	<0.0001	<0.01	0.10	<0.0001
*APOE4*−, *n* (%)	51 (49.5)	70 (63.1)	38 (65.5)	216 (60.2)	3628 (74.9)				
PRS−*APOE4−*	43 (41.7)	63 (56.8)	34 (58.6)	193 (53.8)	3261 (67.3)	<0.0001	0.02	0.44	<0.0001
PRS+*APOE4−*	8 (7.8)	7 (6.3)	4 (6.9)	23 (6.4)	367 (7.6)				
PRS−*APOE4*+	7 (6.8)	9 (8.1)	6 (10.4)	31 (8.6)	372 (7.7)				
PRS+*APOE4*+	45 (43.7)	32 (28.8) (31.5)	14 (24.1)	112 (31.2)	844 (17.4)				

*p* values reported are for comparisons between Alzheimer’s disease^1^, vascular dementia^2^, mixed dementia^3^, and all-cause dementia^4^ cases and participants without dementia diagnoses.

*APOE*, apolipoprotein E *APOE4*+, ≥1 ε4 allele PRS, polygenic risk score.

### AD prediction

After linkage disequilibrium analyses, exclusion of the *APOE* locus including SNPS located directly down or upstream from *APOE*, and further exclusion of SNPs with MAF < 0.01, 72 SNPs reaching genome-wide significance were included in the PRS (Supplementary Table 1**)**. PRS+ and *APOE4*+ participants had 3.40 (95%CI: 2.28–5.09) and 3.34 (95% CI: 2.24–4.99) times the odds of developing AD within 17 years than PRS- and *APOE*-participants, respectively (Table 2). Participants that were both PRS+ *APOE4*+ had a 4.6-fold increased risk in developing AD compared with PRS− *APOE4*− participants (OR, 95% CI: 4.59, 2.96–7.11). Furthermore, increased odds of AD per SD increase of the PRS was evident (OR, 95% CI: 1.70, 1.45–1.99), which remained true even after additionally adjusting for *APOE* status (OR, 95% CI: 1.52, 1.26–1.84). Participants that had one and two *APOE* ε4 alleles had 3- and 14-fold greater odds to be diagnosed with AD compared with participants with two ε3 alleles.

**Table 2:** Logistic regression results of the PRS and *APOE* for Alzheimer’s disease, vascular dementia, mixed dementia, and all-cause dementia.

	Alzheimer’s disease, *n* = 103			Vascular dementia, *n* = 111			Mixed dementia, *n* = 58			All-cause dementia, *n* = 359		
	*n*	OR (95% CI)	*p* value	*n*	OR (95% CI)	*p* value	*n*	OR (95% CI)	*p* value	*n*	OR (95% CI)	*p* value
PRS per SD	103	**1.70 (1.45–1.99)**	**<0.0001**	111	**1.21 (1.01–1.46)**	**0.04**	58	1.16 (0.89–1.52)	0.27	359	**1.36 (1.23–1.51)**	**<0.0001**
PRS per SD^a^	103	**1.52 (1.26–1.84)**	**<0.0001**	111	1.22 (0.98–1.50)	0.07	58	1.02 (0.74–1.40)	0.90	359	**1.30 (1.15–1.47)**	**<0.0001**
PRS−	50	Reference		72	Reference		40	Reference		224	Reference	
PRS+	53	**3.40 (2.28–5.09)**	**<0.0001**	39	**1.65 (1.10–2.47)**	**0.02**	18	1.40 (0.79–2.50)	0.25	135	**1.91 (1.51–2.42)**	**<0.0001**
PRS Q1	19	Reference		29	Reference		18	Reference		81	Reference	
PRS Q2	15	0.74 (0.37–1.49)	0.40	16	0.49 (0.26-0.92)	0.03	14	0.75 (0.36–1.54)	0.43	69	0.79 (0.56–1.11)	0.17
PRS Q3	16	0.84 (0.43–1.66)	0.62	27	0.91 (0.53–1.57)	0.73	10	0.53 (0.24–1.17)	0.12	77	0.93 (0.66–1.29)	0.65
PRS Q4	53	**3.17 (1.84–5.46)**	**<0.001**	39	1.40 (0.85–2.31)	0.19	16	0.98 (0.48–1.98)	0.95	132	**1.80 (1.33–2.44)**	**<0.001**
*APOE4−*	51	Reference		70	Reference		38	Reference		216	Reference	
*APOE4+*	52	**3.34 (2.24–4.99)**	**<0.0001**	41	**1.84 (1.23–2.74)**	**<0.01**	20	**1.75 (1.00–3.07)**	**<0.05**	143	**2.20 (1.74–2.78)**	**<0.0001**
*APOE* ε3ε3	39	Reference		52	Reference		29	Reference		166	Reference	
*APOE* ε3ε4	42	**3.42 (2.17–5.37)**	**<0.0001**	33	**1.94 (1.23–3.05)**	**<0.01**	13	1.46 (0.75–2.87)	0.27	109	**2.09 (1.60–2.73)**	**<0.0001**
*APOE* ε4ε4	8	**13.89 (5.88–32.82)**	**<0.0001**	2	1.88 (0.42–8.36)	0.41	6	**14.81 (5.12–42.87)**	**<0.0001**	18	**6.86 (3.71–12.69)**	**<0.0001**
PRS− *APOE4*−	43	Reference		63	Reference		34	Reference		193	Reference	
PRS+*APOE4*−	8	1.55 (0.71–3.37)	0.27	7	0.90 (0.40–2.00)	0.80	4	0.88 (0.30–2.55)	0.35	23	0.95 (0.60–1.52)	0.84
PRS− *APOE4+*	7	1.42 (0.63–3.23)	0.40	9	1.24 (0.60–2.56)	0.56	6	1.54 (0.63–3.79)	0.81	31	1.44 (0.95–2.19)	0.09
PRS+*APOE4*+	45	**4.59 (2.96–7.11)**	**<0.0001**	32	**2.08 (1.34–3.25)**	**<0.01**	14	1.82 (0.95–3.48)	0.07	112	**2.54 (1.95–3.30)**	**<0.0001**

Model covariates included age, sex, education, and 10 principal components. Bolded results indicate achievement of statistical significance, *p* < 0.05.

*APOE4*+ ≥1 *APOE* ε4 allele, *PRS* polygenic risk score, *Q* quartile, *SD* standard deviation.

^a^Additionally adjusted for *APOE* status.

**Fig. 2:**
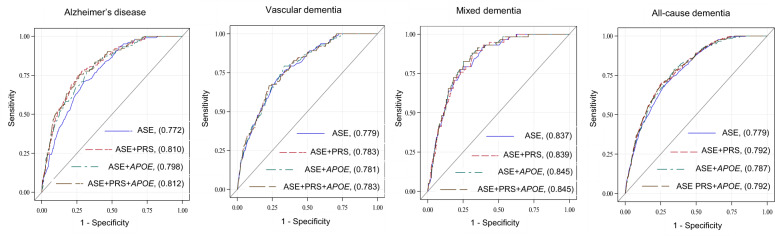
ROC Curves and contrast for Alzheimer’s disease (AD), vascular dementia (VD), mixed dementia (MD), and all-cause dementia (ACD). Predictors included: (1) Age, sex and education (ASE); (2) ASE+PRS (continuous); (3) ASE+*APOE* (categorical); and (4) ASE+PRS+*APOE*. The area under the curve values are reported in the figure. P values are reported for differences between areas under the ROC curves: (1) ASE and ASE+PRS for AD (*p* < 0.01), VD (*p* = 0.40), MD (*p* = 0.67), and ACD (*p* < 0.01). (2) ASE and ASE+*APOE* for AD (*p* = 0.06), VD (*p* = 0.40), MD (*p* = 0.25), and ACD (*p* = 0.02). (3) ASE and ASE+PRS+*APOE* for AD (*p* < 0.01), VD (*p* = 0.41), MD (*p* = 0.29), and ACD (*p* < 0.01).

The addition of the PRS to the base model of age sex and education (ASE) significantly improved AD prediction (Fig. 2, c-statistic: ASE, 0.772; ASE + PRS, 0.810, *p* < 0.01). *APOE* also improved prediction but not significantly (c-statistic: ASE + *APOE*, 0.798, *p* = 0.06).

Stratified analyses and interaction testing based upon age, sex, and education can be found in Table 3. There were no significant interactions, and individuals that were of high genetic risk (PRS+ & *APOE4*+) had similar odds of AD diagnosis regardless of age, sex, or education.

**Table 3:** Dementia diagnoses prediction by PRS & *APOE4* status stratified by sex, age at baseline, and education.

	*n*	PRS + vs. PRS−		*APOE4* + *vs. APOE4−*		PRS+ & *APOE4*+ *vs. PRS− & APOE4−*		Interaction		
								PRS	*APOE4*	PRS&*APOE4*
		OR (95% CI)	*p* value	OR (95% CI)	*p* value	OR (95% CI)	*p* value	*p* value	*p* value	*p* value
Alzheimer’s disease										
Female	60	**3.50 (2.05–5.99)**	**<0.0001**	**3.33 (1.94–5.70)**	**<0.0001**	**4.60 (2.59–8.16)**	**<0.0001**	0.91	0.85	0.99
Male	43	**3.58 (1.91–6.71)**	**<0.0001**	**3.73 (2.00–6.94)**	**<0.0001**	**5.08 (2.51–10.26)**	**<0.0001**			
50–64 years	30	**3.44 (1.66–7.14)**	**<0.001**	**3.77 (1.81–7.84)**	**0.001**	**4.75 (2.13–10.60)**	**0.0001**	0.92	0.75	0.99
65–75 years	73	**3.48 (2.15–5.63)**	**<0.0001**	**3.13 (1.94–5.06)**	**<0.0001**	**4.45 (2.64–7.49)**	**<0.0001**			
≤9 years educ.	85	**3.36 (2.15–5.25)**	**<0.0001**	**3.59 (2.30–5.61)**	**<0.0001**	**4.76 (2.93–7.73)**	**<0.0001**	0.52	0.57	0.81
>9 years educ.	17	**4.67 (1.64–13.31)**	**<0.01**	2.58 (0.92–7.24)	0.07	**4.80 (1.54–14.96)**	**<0.01**			
Vascular dementia										
Female	59	1.73 (0.98–3.05)	0.06	**2.09 (1.20–3.63)**	**<0.01**	**2.19 (1.15–4.18)**	**0.02**	0.74	0.43	0.58
Male	52	1.60 (0.88–2.91)	0.12	1.61 (0.89–2.91)	0.11	**2.00 (1.06–3.75)**	**0.03**			
50–64 years	27	2.17 (0.99–4.75)	0.05	1.77 (0.80–3.91)	0.16	2.26 (0.93–5.46)	0.07	0.59	0.85	0.76
65–75 years	84	1.59 (0.99–2.55)	0.05	**1.93 (1.21–3.05)**	**<0.01**	**2.14 (1.28–3.57)**	**<0.01**			
≤9 years educ.	83	1.41 (0.87–2.29)	0.17	1.43 (0.88–2.31)	0.15	1.57 (0.91–2.70)	0.11	0.24	0.06	0.12
>9 years educ.	23	**2.68 (1.14–6.31)**	**0.02**	**3.64 (1.54–8.57)**	**<0.01**	**4.21 (1.67–10.62)**	**<0.01**			
Mixed dementia										
Female	34	1.01 (0.38–2.69)	0.99	1.73 (0.70–4.28)	0.24	1.23 (0.38–4.05)	0.73	0.37	0.77	0.44
Male	24	1.78 (0.85–3.74)	0.13	1.87 (0.90–3.89)	0.10	**2.32 (1.04–5.17)**	**0.04**			
50–64 years	10	**4.54 (1.25–16.51)**	**0.02**	**4.23 (1.17–15.33)**	**0.03**	**6.08 (1.41–26.19)**	**0.02**	0.02	0.07	0.02
65–75 years	48	1.10 (0.56–2.15)	0.79	1.43 (0.75–2.73)	0.27	1.34 (0.62–2.90)	0.45			
≤9 years educ.	44	1.23 (0.61–2.46)	0.56	1.34 (0.68–2.63)	0.40	1.43 (0.65–3.14)	0.38	0.40	0.04	0.17
>9 years educ.	11	2.05 (0.58–7.21)	0.26	**4.57 (1.24–16.88)**	**0.02**	**4.13 (1.02–16.80)**	**<0.05**			
All-cause dementia										
Female	182	**1.87 (1.33–2.63)**	**<0.001**	**2.16 (1.54–3.04)**	**<0.0001**	**2.45 (1.67–3.60)**	**<0.0001**	0.78	0.68	0.75
Male	177	**2.05 (1.47–2.86)**	**<0.0001**	**2.32 (1.67–3.22)**	**<0.0001**	**2.74 (1.91–3.94)**	**<0.0001**			
50–64 years	95	**2.36 (1.56–3.59)**	**<0.0001**	**2.63 (1.74–3.98)**	**<0.0001**	**3.03 (1.92–4.79)**	**<0.0001**	0.40	0.32	0.35
65–75 years	264	**1.78 (1.35–2.36)**	**<0.0001**	**1.97 (1.49–2.60)**	**<0.0001**	**2.30 (1.69–3.14)**	**<0.0001**			
≤9 years educ.	279	**1.83 (1.39–2.41)**	**<0.0001**	**1.98 (1.51–2.59)**	**<0.0001**	**2.32 (1.72–3.14)**	**<0.0001**	0.42	0.10	0.25
>9 years educ.	66	**2.22 (1.30–3.77)**	**<0.01**	**2.97 (1.74–5.06)**	**<0.0001**	**3.31 (1.84–5.93)**	**<0.0001**			

All analyses stratified by 10 principal components and: sex were adjusted for age and education, age for sex and education, and education for sex and age. Bolded results indicate achievement of statistical significance, *p* < 0.05.

*APOE4* apolipoprotein E ε4, *PRS* polygenic risk score, *Educ*. education.

### VD prediction

The PRS and *APOE* were predictive of VD (OR, 95% CI: PRS+: 1.65, 1.10–2.47; *APOE4*+: 1.84, 1.23–2.74; PRS+ *APOE4*+: 2.08, 1.34–3.25) (Table 2). The genotype *APOE* ε3ε4 was associated with twofold greater odds of VD when compared with the reference group *APOE* ε3ε3. ROC curve analysis revealed no significant differences in prediction by the addition of the PRS or *APOE* to age, sex, and education (Fig. 2).

The stratified analyses revealed no significant interactions between AD genetic risk and age, sex, and education in the prediction of VD diagnosis (Table 3).

### MD prediction

The PRS was not predictive of MD diagnosis; however, *APOE4*+ was predictive of MD diagnosis (OR, 95% CI: 1.75, 1.00–3.07) and participants that were *APOE* ε4ε4 compared with *APOE* ε3ε3 participants had 15-fold increased risk of diagnosis (OR, 95% CI: 14.81, 5.12–42.87). ROC curve analysis revealed no significant differences in prediction by the addition of the PRS or *APOE* to age, sex, and education (Fig. 2). Stratified analyses revealed an interaction between age and AD genetic risk, however a limited number of cases were included in the analysis (Table 3).

### All-cause dementia prediction

PRS and *APOE4* status were significantly predictive of all-cause dementia (Table 2). PRS+ and *APOE4*+ participants each had increased odds of all-cause dementia diagnosis (OR, 95% CI: PRS+: 1.91, 1.51-2.42; *APOE4*+: 2.20, 1.74–2.78). Participants that were PRS+*APOE4*+ expressed 2.5-fold increased odds of all-cause dementia. One SD increase in the PRS resulted in 1.4-fold greater odds of dementia diagnosis (OR, 95% CI: 1.36, 1.23–1.51). In addition, participants with one and two *APOE* ε4 alleles had two- and seven-fold increased odds of all-cause dementia compared with participants with two ε3 alleles.

ROC curve analysis illustrated all-cause dementia prediction was significantly improved when adding the PRS (c-statistic: 0.792, *p* < 0.01), *APOE* (c-statistic: 0.787, *p* = 0.02) and the PRS & *APOE* (c-statistic: 0.792, *p* < 0.01) to age, sex, and education (c-statistic: 0.779).

There were no significant interactions between AD genetic risk and age, sex, and education in the prediction of dementia diagnosis (Table 3).

## Discussion

In a prospective community-based cohort independent of the IGAP consortia, PRS positivity expressed significant predictive ability of AD diagnosis beyond *APOE* status, with stronger associations to AD than VD, MD, or all-cause dementia. Participants that were both PRS and *APOE4* positive exhibited 4.6-fold greater odds of AD diagnosis within 17 years compared with participants who were both PRS and *APOE4* negative.

Our PRS builds upon a rich foundation of previous AD PRSs, with nearly twenty studies expressing significant ability of the PRS to discern AD [8, 34, 35]. Four of these studies have also utilized a cohort approach: (1) Chouraki et al. used eight prospective cohorts from the IGAP consortia, a mix of varying types of cohort studies including the Rotterdam study; [36] (2) Tan et al. examined the PRS in a clinical cohort; [37] and (3) finally two studies by Ahmad et al. and Van der Lee et al. utilized the community-based Rotterdam cohort study [38, 39]. Our community-based cohort was however the only study completely independent of previous GWA meta-analyses from which the PRSs were derived and that utilized the most recent IGAP data. Community-based cohorts play an implicit role in contribution to the study of risk factor-outcome associations [40] and are important in establishing the future role of PRSs in genetic risk stratification.

Interestingly, in the Rotterdam cohort study Van der Lee at al. reported similar likelihood of AD or dementia development based upon the PRS (HR, 95% CI: AD, 1.11, 0.97–1.27; dementia, 1.11, 0.99–1.26) [39]. No other studies considered prediction of all-cause dementia and none VD/MD. In our study, we found the PRS to be more predictive of AD than VD, MD, and all-cause dementia. A large proportion of all-cause dementia cases included AD cases in our study, which could explain the association between AD genetic risk and all-cause dementia. This could also be the reason for the similar associations found between the PRS and AD, and the PRS and dementia in the Rotterdam study [39].

Often AD diagnosed patients additionally exhibit cerebrovascular pathology and VD diagnosed patients have evident AD pathology, which may go undiagnosed as mixed dementia [41]. This should be taken into account when considering clinical diagnoses of dementia and could additionally account for the ability of our PRS to predict VD and all-cause dementia. However, the much larger associations between the PRS and AD compared with the other dementia subtypes supports the specificity of AD genetic risk and heterogeneity of the genetic architecture among dementia subtypes. Larger independent cohort studies are necessary to explore the relationship between AD genetic risk and other dementia subtypes for more insight into the underlying genetic architecture and mediating influence of genetics.

There was a lack of significant interaction between AD genetic risk and age, sex, and education in the prediction of AD, VD, MD, and all-cause dementia diagnoses. This supports the idea that prediction of dementia based upon AD genetic risk is similar regardless of these important AD risk factors. The case numbers for several categories were however rather small and should be interpreted with caution.

### Implications

Presently, the utilization of a PRS in addition to *APOE4* could be used to enhance genetic risk stratification as it provides additional genetic information and greater AD prediction ability. PRSs in clinical trials could be used to target individuals who may be at risk for AD before any pathological changes occur in the brain, which is critical in the search for a successful therapy preventing AD.

In the future, PRSs for AD may play a paramount role in precision medicine with targeted therapies based upon AD genetic risk. Enhanced genetic risk stratification could also help identify the best candidates for AD preventive treatment, before accumulation of amyloid in the brain or for individualized treatments based upon genetic make-up. Recently, it has been shown that the effects of lifestyle behaviors are mediated by genetic risk in dementia development [42]. A multi-domain intervention approach involving modifiable vascular and lifestyle risk factors, which has shown to improve/maintain cognitive function in older adults, could be recommended based on genetic risk [43].

### Strengths and weaknesses

The greatest strengths of our study is the epidemiological approach to the investigation of a PRS for AD in a large community-based cohort prospectively followed over 17 years that is completely independent of the IGAP consortia, and the novel use of the latest GWA meta-analysis data. In addition, we investigated VD/MD diagnoses and completed age, sex, education stratified analyses, which no other study has addressed.

There are however several limitations including the possibility of dementia misdiagnosis/underdiagnosis. The dementia diagnoses made in the ESTHER study were clinical diagnoses reported heterogeneously by numerous practitioners, and may be inferior to diagnostic standards that can be achieved in highly specialized academic settings. This is however the nature of community-based cohort studies, which portray common practice in such a setting. In addition, dementia neuropathologies are complex where AD pathology seldom occurs in isolation[19], further complicating diagnoses. Only 63% of participants with available genetic information also had dementia information available in our cohort. Although an inherent characteristic of prospective cohort studies, non-response bias may have led to an underestimation of dementia. The AD, MD, and VD case numbers were rather small, especially in the stratified analysis, which led to large CIs and a general lack of power. In addition, the participants without dementia diagnosis were significantly younger at baseline which could have led to missed dementia diagnoses that would have been made at higher ages. Finally, this study has limited generalizability as its population consisted of participants of European descent.

## Conclusion

A PRS encompassing additional genetic variants derived from the most current AD GWA meta-analysis enriched the ability of *APOE* status to discern AD in a prospective community-based cohort followed over 17 years that was independent of previous GWA meta-analyses. The PRS expressed a greater ability to predict AD than VD, MD, or all-cause dementia. Therapeutic treatment development and eventually precision medicine could benefit from enhanced risk stratification through the utilization of an AD PRS in addition to *APOE* status.

## Supplementary information

Supplementary Table 1, Supplementary Table 2, Supplementary Table 3
