# Supplementary material for: Prediction of clinical diagnosis of Alzheimer’s disease, vascular, mixed, and all-cause dementia by a polygenic risk score and APOE status in a community-based cohort prospectively followed over 17 years
Source: Mol Psychiatry. 2020 May 13;26(10):5812–22. doi: 10.1038/s41380-020-0764-y (PMC8758470; doi:10.1038/s41380-020-0764-y)
Supplement: Supplementary file 1 — Supplementary Table 1, Supplementary Table 2, Supplementary Table 3 [file 41380_2020_764_MOESM1_ESM.docx]

| **Supplementary Table 1: SNPs included PRS** | | | |
| --- | --- | --- | --- |
| MarkerName | Gene-variant type | MarkerName | Gene-variant type |
| rs620807 | AC005779.2, AC006126.3, MARK4- intron, intron, intron | rs10838724 | CELF1-intron |
| rs12460985 | AC005779.2, MARK4-3 prime UTR, intron | rs144645090 | CKM, intron |
| rs346739 | AC006126.3, MARK4-intron, intron | rs1968449 | CLASRP, RELB-intron, downstream |
| rs2627642 | AC006126.3,AC005779.2, MARK4- intron, intron, intron | rs112481437 | CLASRP, RNU6-611P-intron, upstream |
| rs4147904 | AC011558.5, CNN2, ABCA7- upstream gene, downstream gene, intron | rs145639166 | CLPTM1-intron |
| rs60049679 | APOC1, APOC1P1-intergenic, upstream | rs7257916 | CLPTM1-intron |
| rs144311893 | APOC1-downstream | rs11118328 | CR1L-intron |
| rs12691088 | APOC1-intron | rs75178253 | CTB-129P6.11, CLPTM1-upstream, intron |
| rs3925681 | APOC1-intron | rs76692773 | CTB-129P6.4, PVRL2, TOMM40-upstream, downstream, intron |
| rs73033507 | APOC1P1-intron | rs71352238 | CTB-129P6.4, PVRL2, TOMM40-upstream, downstream, intron |
| rs8106813 | APOC1P1-intron | rs73936968 | CTB-129P6.4, PVRL2, TOMM40-upstream, downstream, intron |
| rs12721109 | APOC4, APOC2- intron, upstream | rs11668861 | CTB-129P6.4, PVRL2-downstream gene, intron |
| rs1132899 | APOC4, APOC2- missense, upstream | rs79701229 | CTB-129P6.4, PVRL2-downstream gene, intron |
| rs56394238 | BCAM-intergenic | rs157590 | CTB-129P6.4, TOMM40-upstream gene, intron |
| rs1135062 | BCAM-missense | rs2289494 | CTB-171A8.1, CEACAM19-intron, 3 prime UTR |
| rs4803748 | BCL3, snoZ6-upstream gene, intergenic | rs111358663 | CTB-171A8.1-intron |
| rs62117206 | BCL3-intron | rs346758 | EXOC3L2, MARK4- intron, intron |
| rs6733839 | BIN1-intergenic | rs62118504 | EXOC3L2, MARK4- intron, intron |
| rs6431223 | BIN1-intergenic | rs4147929 | HMHA1, ABCA7-upstream gene, intron |
| rs10929006 | BIN1-intron | rs580064 | MS4A2-intergenic |
| rs13032148 | BIN1-upstream | rs8108110 | snoZ6-upstream gene |
| rs3135348 | BTNL2-intergenic | rs11218343 | SORL1-intron |
| rs2889414 | CBLC-intron | rs3740688 | SPI1-missense |
| rs2965112 | CBLC-intron | rs118170342 | TOMM40-intron |
| rs11669005 | CEACAM22P-intron | rs1160984 | TOMM40-intron |
| rs611267 | MS4A4E-intron | rs114812713 | OARD1, APOBEC2-3 prime UTR, downstream |
| rs617135 | MS4A6A, AP001257.1-downstream, intergenic | rs11762262 | EPHA1-AS1, EPHA1-intron, upstream |
| rs4752801 | NUP160-intergenic | rs2279590 | CLU-intron |
| rs7255066 | PVR, CTB-171A8.1- upstream gene, intron | rs755951 | PTK2B-intron |
| rs41289512 | PVRL2-intron | rs9381563 | AL355353.1-upstream |
| rs12974942 | PVRL2-intron | rs12590654 | SLC24A4-intron |
| rs57537848 | PVRL2-intron | rs3844143 | snoU13-intergenic |
| rs10410835 | PVRL2-intron | rs35194062 | RELB-intron |
| rs365653 | PVRL2-intron | rs874743 | RELB-intron |
| rs146275714 | PVRL2-intron | rs28697575 | RELB-intron |
| rs412776 | PVRL2-intron | rs12416487 | RP11-138I18.2-upstream gene |

| Supplementary Table 2: Participant *APOE* characteristics - ESTHER cohort study | | | | | | | | |  |
| --- | --- | --- | --- | --- | --- | --- | --- | --- | --- |
|  | Alzheimer’s  Disease | Vascular  Dementia | Mixed  Dementia | All-cause Dementia | Participants  without dementia | p-value^1^ | p-value^2^ | p-value^3^ | p-value^4^ |
| n | 103 | 111 | 58 | 359 | 4844 |  |  |  |  |
| *APOE* ε2ε2, n (%) | 1 (1.0) | 0 | 0 | 1 (0.3) | 37 (0.8) | <.0001 | .08 | <.0001 | <.0001 |
| *APOE* ε2ε3, n (%) | 11 (10.7) | 18 (16.2) | 9 (15.5) | 49 (13.7) | 726 (15.0) |  |  |  |  |
| *APOE* ε2ε4, n (%) | 2 (1.9) | 6 (5.4) | 1 (1.7) | 16 (4.5) | 166 (3.4) |  |  |  |  |
| *APOE* ε3ε3, n (%) | 39 (37.9) | 52 (46.9) | 29 (50.0) | 166 (46.2) | 2865 (59.2) |  |  |  |  |
| *APOE* ε3ε4, n (%) | 42 (40.8) | 33 (29.7) | 13 (22.4) | 109 (30.4) | 986 (20.4) |  |  |  |  |
| *APOE* ε4ε4, n (%) | 8 (7.8) | 2 (1.8) | 6 (10.3) | 18 (5.0) | 64 (1.3) |  |  |  |  |

Note: *p*-values reported are for comparisons between Alzheimer’s disease^1^, vascular dementia^2^, mixed dementia^3^, and all-cause dementia^4^ cases and participants without dementia

| **Supplementary Table 3: IGAP Support and Funders** |
| --- |
| IGAP was made possible by the generous participation of the control subjects, the patients, and their families. The i–Select chips was funded by the French National Foundation on Alzheimer's disease and related disorders. EADI was supported by the LABEX (laboratory of excellence program investment for the future) DISTALZ grant, Inserm, Institut Pasteur de Lille, Université de Lille 2 and the Lille University Hospital. GERAD/PERADES was supported by the Medical Research Council (Grant n° 503480), Alzheimer's Research UK (Grant n° 503176), the Wellcome Trust (Grant n° 082604/2/07/Z) and German Federal Ministry of Education and Research (BMBF): Competence Network Dementia (CND) grant n° 01GI0102, 01GI0711, 01GI0420. CHARGE was partly supported by the NIH/NIA grant R01 AG033193 and the NIA AG081220 and AGES contract N01–AG–12100, the NHLBI grant R01 HL105756, the Icelandic Heart Association, and the Erasmus Medical Center and Erasmus University. ADGC was supported by the NIH/NIA grants: U01 AG032984, U24 AG021886, U01 AG016976, and the Alzheimer's Association grant ADGC–10–196728. |
